# Tick-Borne Pathogens, *Babesia* spp. and *Borrelia burgdorferi* s.l., in Sled and Companion Dogs from Central and North-Eastern Europe

**DOI:** 10.3390/pathogens11050499

**Published:** 2022-04-21

**Authors:** Anna Bajer, Maciej Kowalec, Viktoriya A. Levytska, Ewa Julia Mierzejewska, Mustafa Alsarraf, Vasyl Poliukhovych, Anna Rodo, Dagmara Wężyk, Dorota Dwużnik-Szarek

**Affiliations:** 1 Department of Eco-Epidemiology of Parasitic Diseases, Institute of Developmental Biology and Biomedical Sciences, Faculty of Biology, University of Warsaw, Miecznikowa 1, 02-096 Warsaw, Poland; kowalec.maciej@gmail.com (M.K.); ewajuliamierzejewska@gmail.com (E.J.M.); elmusto0@gmail.com (M.A.); d.wezyk@student.uw.edu.pl (D.W.); dorota.dwuznik@biol.uw.edu.pl (D.D.-S.); 2 Department of Infection and Invasive Diseases, Faculty of Veterinary Medicine and Technology in Animal Husbandry, State Agrarian and Engineering University in Podilia, 32300 Kamianets-Podilskyi, Ukraine; levytska28@gmail.com (V.A.L.); vasiapoluhovich.vet@gmail.com (V.P.); 3 Department of Pathology and Veterinary Diagnostics, Warsaw University of Life Sciences—SGGW, 02-766 Warsaw, Poland; kazanekr@wp.pl

**Keywords:** *Babesia canis*, Borrelia burgdorferi, paralogs, sled dog, Lithuania, Latvia, Estonia, Poland, Ukraine, PCR

## Abstract

Ticks are important vectors of numerous pathogens of medical and veterinary significance. The aim of the current study was to determine the prevalence of *Babesia* spp. and *Borrelia burgdorferi* s.l. in sled and pet dogs from Central and North-Eastern Europe. Neither *Babesia* spp. nor *Borrelia burgdorferi* s.l. infections were detected in sled dogs from seven countries (Poland, Lithuania, Latvia, Estonia, Belarus, Russia and Finland). The DNA of *Babesia* spp. was detected in 100% of symptomatic and 5.4% of asymptomatic pet dogs from Poland. Similarly, the DNA of *Babesia* spp. was identified in 82% of symptomatic and 3.8% of asymptomatic pet dogs from Ukraine. The DNA of *Borrelia burgdorferi* s.l. was detected in 4.4% of pet dogs. Molecular typing confirmed the presence of *Babesia canis* and *Borrelia burgdorferi* sensu stricto (s.s.) in selected samples. Four dogs were co-infected by *B. canis* and *Borrelia burgdorferi* s.l. Tick-borne pathogens constitute a serious health threat to pet dogs in Central and South-Eastern Europe, but were not observed among sled dogs from the same region of Europe nor in the Baltic countries.

## 1. Introduction

Ticks are important vectors of numerous microorganisms of medical and veterinary significance [1,2]. Among tick-borne pathogens (TBP) with the greatest impact on their hosts are spirochaetes of the *Borrelia burgdorferi sensu lato* (s.l.) complex, responsible for development of the multisystemic disease, borreliosis (Lyme disease, Lyme borreliosis; LB). Globally, borreliosis affects between 0.3–0.5 million people/year in the Northern Hemisphere and in the next decade the incidence of Lyme disease may increase by 20% in the USA only as a consequence of climate change [3,4]. In Poland in 2019, 20,630 cases of Lyme disease were registered, and 1701 people were hospitalized. The highest incidence of 107.7/100,000 population was recorded in the Podlaskie voivodeship, for many years the province in the country with the highest incidence of borreliosis [5]. Such a high incidence in the human population is associated with the high prevalence of spirochaetes (10–25%) in its main vector, *Ixodes ricinus* ticks [4]. Borreliosis also affects dogs [6,7]. The most common clinical manifestation of Lyme disease in dogs is Lyme arthritis. The classical presentation is an acute monoarticular or polyarticular lameness with joint swelling, fever, lethargy, and mild local lymphadenopathy, usually in young, often large-breed dogs with an active outdoor lifestyle [8]. Rare manifestation is Lyme carditis, Lyme nephritis and Lyme neuritis [7,8,9,10]. Clinical LB cases are more common in the US than in Europe [8]. Subclinical infections are the most common [8,10,11].

*Babesia* spp. are protozoan parasites of red blood cells responsible for the development of babesiosis, a potentially life-threatening disease of humans and animals [12,13]. Although cases of babesiosis are rare in humans in Europe [14], the symptoms may resemble malaria infection, with hemolytic anemia, hemoglobinuria, and Severe Acute Respiratory Syndrome (SARS) development, and babesiosis can be fatal especially in immunocompromised patients [12].

Frequent clinical signs associated with canine babesiosis are apathy, weakness, anorexia, pale mucous membranes, and a poor general condition [13]. Infection can cause fever, enlarged lymph nodes and spleen, anaemia, thrombocytopenia, jaundice, and pigmenturia. Babesiosis due to *B. canis* can manifest with a range of clinical signs and laboratory abnormalities: petechiae, epistaxis, vomiting, lymphadenomegaly, hypotension, low T3 syndrome, mild to moderate nonregenerative, normochromic and normocytic anaemia, regenerative anaemia (less common), leukopenia with neutropenia and/or lymphopenia, hypoalbuminemia, elevation of liver enzymes (ALT, AST, ALP), hypokalemia, hyponatremia, hyperchloremia, hyperlactatemia, hyperphosphatemia [13]. Babesiosis due to *Babesia canis* infection is an emerging tick-borne disease in dogs in Central Europe [13,15,16,17,18,19]. The main, if not the only, vector of *B. canis* is the ornate dog tick, *Dermacentor reticulatus* [13,20,21].

We have focused here on sled dogs because they are particularly prone to vector-borne infections through their participation in training sessions and racing events, the majority of which take place in forest areas where ticks and mosquitoes are abundant. These dogs are usually housed in kennels where there may be an additional high risk of vector challenge. Infections with TBP may have negative impacts on the performance of sled dogs in competitive events and are therefore of considerable concern for sled dog owners and the organizers of race meetings [22,23]. Although reports on tick-borne infections in dogs from NE Europe, including the Baltic states, are still rare [24,25,26], we have reported recently infections with several TBP in sled dogs, including *Anaplasma phagocytophilum, B. canis*, and tick-borne encephalitis virus (TBEV) [22,23,27,28]. Moreover, high prevalence of *Dirofilaria repens*, a zoonotic mosquito-transmitted nematode, has been found recently in sled dogs from central and NE Europe [29], suggesting an increasing significance of vector-borne infections in these regions of Europe.

The aim of the current study was to determine the prevalence of *Babesia* spp. and *Borrelia burgdorferi* s.l. in sled and pet dogs from Central and North-Eastern (NE) Europe. 

## 2. Materials and Methods

### 2.1. Ethics Approval and Consent to Participate

Since the study was carried out on blood samples provided for veterinary diagnostic purpose [29], no ethical approval/license was required for our study (as per resolution on the protection of animals used for scientific or educational purposes, 15th January 2015 [Dz. U. 2015 position 266] Chapter 1, Paragraph 1.2.1).

### 2.2. Collection of Samples

Blood samples from dogs were collected in 2015–2021, with most samples collected between 2017–2019. Five hundred and fifty-three dog blood samples were collected into EDTA-covered vials from eight countries, including 256 samples from various breeds of sled dogs (mainly Alaskan huskies, Siberian huskies, and European sled dogs [ESD]), and 297 samples were from pet dogs (Table 1). The main study was focused on sled dogs from Poland and the Baltic countries (Lithuania, Latvia, and Estonia) and on companion dogs from Poland and Ukraine (Table 1). Additionally, several sled dogs were available for sampling from Russia, Belarus, and Finland (Table 1).

All sled dogs were healthy animals, comprising racing dogs and a small group of elderly individuals, living in the same kennels. Sled dogs were sampled in their kennels or during national or international sled dog races in 2017–2021, encompassing dogs from Poland, Lithuania, Latvia, Estonia, Belarus, Russia, and Finland. Basic data were noted, including breed, age, sex, and health status (i.e., activity level, appetite, and any health or stamina problems, as reported by the owners).

Blood samples from pet dogs originating from Central Poland and Western Ukraine were from animals with laboratory-confirmed babesiosis (68 from Poland and 50 from Ukraine) and also from a diverse group of dogs attending veterinary clinics for different non-TBD-related concerns. Additionally, we sampled a diverse group of healthy pet dogs presenting no signs of TBDs (owned pedigree dogs and mixed breed dogs from a shelter) from a single location (Błędowo near Nowy Dwór Mazowiecki, Central Poland) (Table 1).

### 2.3. Molecular Detection of Babesia spp. and Borrelia burgdorferi Sensu Lato

Genomic DNA was extracted from EDTA-preserved blood samples using the DNAeasy Blood & Tissue kit (Qiagen, Hilden, Germany) and stored at a temperature of −20 °C, no longer than six months before PCR testing.

Molecular detection of *Babesia* spp. was performed by amplification of a 550 bp fragment of 18S rDNA, as described previously [30,31,32]. For molecular screening of spirochaetes (*Borrelia burgdorferi* s.l.) genus-specific primers: 132f/905r and 220f/824r were used to amplify the *flaB* gene fragments (774 and 605 bp), respectively [33], in a nested-PCR protocol using modified reaction conditions [4]. Positive (sequenced isolates of *Babesia microti* or *Borrelia burgdorferi* s.l.) and negative (sterile water) controls were incorporated in each set of PCRs. Amplicons were visualized with Midori Green stain (Nippon Genetics Europe GmbH, Düren, Germany) following electrophoresis in 1.5% agarose gels. For the identification of pathogen species, selected amplicons were purified and sequenced in both directions by a private company (Genomed S.A., Warsaw, Poland). DNA sequence alignments were conducted using MEGA X (https://www.megasoftware.net/ (accessed on 5 March 2022)) and CodonCode Aligner. The resulting consensus sequences were compared with sequences deposited in GenBank NCBI.

For the statistical evaluation of differences in pathogen prevalence (% infected), we applied maximum likelihood techniques based on log linear analysis of contingency tables in the IBM SPSS Statistics: PS IMAGO PRO Academic v.7 (institutional license purchased by the University of Warsaw, Warsaw, Poland), as described in detail in our previous papers [15,23]. Country of dog origin (eight levels), sex of dogs (males and females), sled dog status (0, 1) and *Babesia* infection status (veterinary laboratory result) (0, 1) were used as the factors in models with the presence or absence of *Babesia* DNA considered as a binary factor (0, 1). For each level of analysis in turn, beginning with the most complex model, involving all possible main effects and interactions, those combinations that did not contribute significantly (*p* > 0.05) to explaining variation in the data were eliminated in a stepwise fashion beginning with the highest-level interaction (backward selection procedure). A minimum sufficient model was then obtained, for which the likelihood ratio of chi-square was not significant, indicating that the model was sufficient in explaining the data [15,23].

Phylogenetic analysis was performed using the Maximum Likelihood method and Tamura 3-parameter model. The evolutionary model was chosen in accordance with the data following implementation of a model test in MEGA X. A discrete Gamma distribution was used to model evolutionary rate differences among sites (5 categories (+G, parameter = 0.2939)). *Borrelia miyamotoi* was selected as outgroup.

Selected *B. canis* and *Borrelia burgdorferi* sequences originating from Poland and Ukraine have been deposited in the GenBank database under the accession numbers: OM350211- OM350213 for *B. canis* from Poland, OM362458-OM362460 for *B. canis* from Ukraine and OM387038-OM387040 for *Borrelia burgdorferi* s.s. from Poland.

## 3. Results

Neither *Babesia* spp. nor *Borrelia burgdorferi* s.l. infections were detected in any of the sled dogs sampled from seven countries (Poland, Lithuania, Latvia, Estonia, Belarus, Russia and Finland) (*Babesia*-positive x sled dog status: χ^2^_1_ = 94.2, *p* < 0.001; *Borrelia*-positive x sled dog status: χ^2^_1_ = 16.4, *p* < 0.001).

The DNA of *Babesia* spp. was detected in 100% of symptomatic and 5.4% of asymptomatic pet dogs from Poland (Table 1). Similarly, *Babesia* DNA was identified in 82% of symptomatic and 3.8% of asymptomatic pet dogs from Ukraine (*Babesia*-positive x *Babesia*-laboratory result: χ^2^_1_ = 427.3, *p* < 0.001; *Babesia*-positive x country of origin: χ^2^_7_ = 14.7, *p* = 0.04). The most common clinical signs observed in symptomatic dogs were lethargy, inappetence and fever (>80% of dogs).

Sequencing of 14 PCR products from dogs from Poland (11 from symptomatic and 3 from asymptomatic dogs) revealed *B. canis* infection (99.6–100% identity with *B. canis* isolates from dogs and red foxes; accession numbers MN173223 and MN134074). Similarly, sequencing of 19 amplicons from dogs from Ukraine confirmed *B. canis* infection (99.4–100% identity with *B. canis* isolates from dogs and red foxes; accession numbers MN134074, MN173223 and MN704759).

Inspection of 18S rDNA chromatograms of *B. canis* sequences from dogs from Poland and Ukraine revealed the occurrence of paralogs as defined by Hrazdilova et al. [34]. Double peaks in core positions (positions 609–610 of full-length 18S rRNA gene of the sequence AY072926) were present in 11 Polish and 9 Ukrainian *B. canis* sequences (Figure S1).

Among the 14 Polish samples, in 3 cases clean chromatograms were obtained, including 2 sequences with GA nucleotides in core position (previous genotype A; [35], Figure S1) and 1 sequence with AG nucleotides (previous genotype B; [35]). Eleven other sequences showed double peaks (presence of different paralogs) in these positions (Figure S1).

Among 19 Ukrainian sequences, in 2 samples a clean chromatogram with GA nucleotides (previous genotype A; [35]) was observed; in 8 samples AG nucleotides (previous genotype B; [35]) were observed, while 9 other sequences showed double peaks (presence of different paralogs) in these positions (Figure S1). Only sequences presenting ‘clean’ chromatograms were deposited in the GenBank database.

The DNA of *Borrelia burgdorferi* s.l. was detected in 13/297 pet dogs (4.4%) (*Borrelia*-positive x sled dog status: χ^2^_1_ = 16.4, *p* < 0.001) (Table 1). In Poland, *Borrelia burgdorferi* s.l. was found in 4.2% of pet dogs, including 4.4% of dogs with babesiosis and 4.1% of healthy pet dogs. In Ukraine, the DNA of *Borrelia burgdorferi* s.l. was detected in only seven ‘healthy’ pet dogs (6.7%) (*Borrelia*-positive x *Babesia*-laboratory result x country of origin: χ^2^_1_ = 3.94, *p* = 0.047). Interestingly, *Borrelia burgdorferi* DNA was detected in four dogs with *B. canis* infection, confirmed by molecular typing (one asymptomatic from Ukraine, three with babesiosis from Poland) (*Babesia*-positive x *Borrelia*-positive: not significant; NS). Furthermore, the DNA of *Di. repens* was identified in these three pet dogs from Poland co-infected by *B. canis* and *Borrelia burgdorferi* s.l. resulting in triple concurrent infections (results of testing for *Dirofilaria* spp. from [29]).

**Figure 1:**
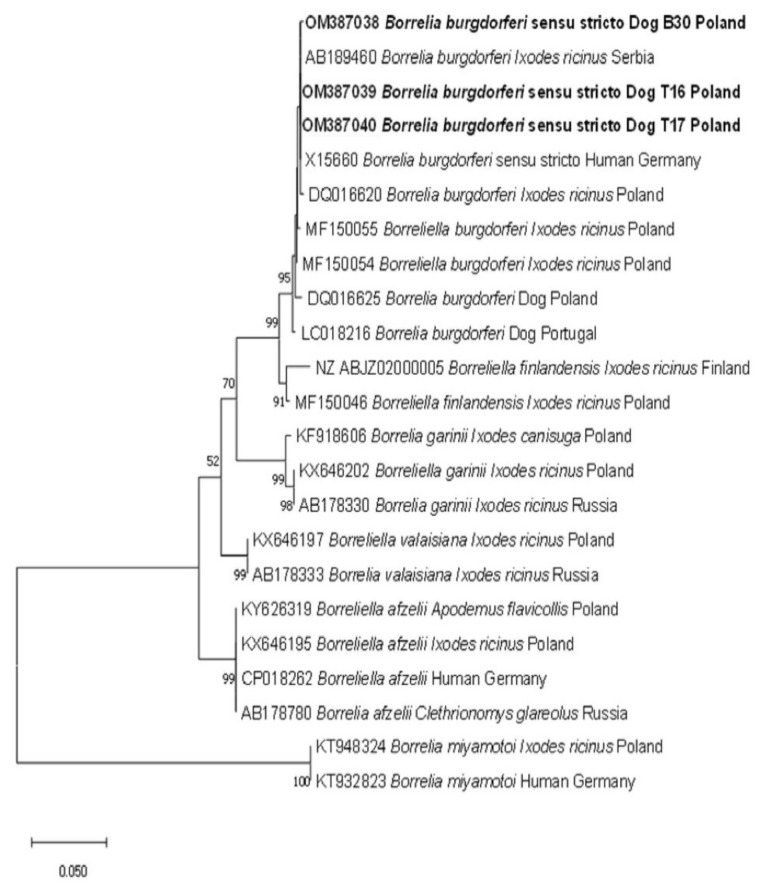
Molecular phylogenetic analysis of *flaB* gene fragment (605 bp) of *Borrelia burgdorferi* s.l. The evolutionary history was inferred by using the Maximum Likelihood method and Tamura 3-parameter model. The tree with the highest log likelihood (−1385.02) is shown. The percentage of trees in which the associated taxa clustered together is shown next to the branches. The tree is drawn to scale, with branch lengths measured in the number of substitutions per site. This analysis involved 23 nucleotide sequences. There was a total of 476 positions in the final dataset.

The sequencing of three *Borrelia burgdorferi* s.l. products resulted in 99.81–100% identity to *Borrelia burgdorferi* sensu strico (s.s.) (MH807139) isolated from an *Ixodes ariadnae* nymph from Poland. Identification of *Borrelia burgdorferi* s.s. was confirmed by the topology of the phylogenetic tree, with our three sequences grouped with other *Borrelia burgdorferi* s.s. sequences (Figure 1).

## 4. Discussion

The main finding of the current study is that tick-borne pathogens, especially *Babesia*, and to lesser extent *Borrelia burgdorferi* s.l., constitute a serious health threat to pet dogs in Central (Poland) and South-Eastern (Ukraine) Europe, but neither pathogen was observed among sled dogs from the same region of Europe nor in those from the Baltic countries.

In the current study, 33 of 117 *Babesia*-positive samples (28%) were sequenced (14 from Poland, 19 from Ukraine); all resulted with identification of *B. canis*. It is in agreement with previous studies, because *B. canis* is reported almost exclusively as the etiological agent of canine babesiosis in Poland [15,17,22,23,27,28], with only four cases of *B. gibsoni* identified recently in the South-Eastern region reviewed in [17]. Our sequencing results are also in agreement with the results of commercial laboratory results. We selected for genotyping samples both with laboratory-confirmed *B. canis* and asymptomatic infections from Poland and Ukraine to determine the exact species involved. We also genotyped positive samples from American Pit Bull Terriers from Ukraine as this breed seems predisposed to *B. gibsoni* infection [17]. All sequenced samples were identified as *B. canis*; thus, it is likely this species is the most common in pet dogs from Central Poland and Western Ukraine, but further studies are needed to genotype more *Babesia*-positive samples.

Our failure to detect *Babesia* and *Borrelia* in sled dogs contrasts with the expected/predicted increased risk of exposure of these animals to vectors, compared to that of pet dogs. However, the majority of sled dogs involved in the current study were strict racing dogs, participating actively in races and training sessions. In such dogs, any health problems are likely to be easily noticed (for example by comparison with the attitudes/performance of other team members), especially regarding exercise intolerance (babesiosis and borreliosis) or joint problems (borreliosis). Furthermore, practically all the reviewed sled dog owners reported the use of prophylactic measures against ticks and tick-borne diseases, as reported in our previous studies [23,27,28].

Interestingly, even sled dogs originating from countries/regions endemic for babesiosis and borreliosis (Poland, Ukraine, Lithuania, and Latvia) were less infected than pet dogs from Poland and Ukraine. This is actually evidence for the good quality of health care that sled dogs receive these days. Interestingly, in military working dogs in the USA, seroprevalence of anti-*Borrelia burgdorferi* antibodies was much lower (0.9%, [36]) than in the general population (1.2–16%, [37,38]).

Our study was based on the molecular detection of microorganisms’ DNA in canine blood samples. The vast majority of published studies, however, are based on the detection of antibodies against TBPs, and hence reflect overall exposure risk, rather than currently present infections [36,37,38]. Serological tests are widely used for the diagnostics of *Borrelia burgdorferi* infection in dogs [37,38,39]. Seroprevalence of anti-*Borrelia burgdorferi* s.l antibodies differs profoundly in dogs from different countries and regions. In two large epidemiological studies from the USA, encompassing 2010–2017 and 2013–2019, seroprevalence ranged between 1.2–16% depending on the year and the state [37,38]. Interestingly, although prevalence decreased in old endemic regions, borreliosis was shown to be expanding in new regions with increasing prevalence in dog populations [37,38]. In a recent study in Canada, seroprevalence was much lower, about 2–3% [40]. 

In Europe, epidemiological studies have revealed a similar discrepancy in seroprevalence of borreliosis between countries/regions and dog breeds/groups [39,41,42,43]. The highest seroprevalence was noted in two groups of Bernese Mountain Dogs from Austria, including healthy dogs and dogs with renal disease (43 and 54%, respectively) [43]. Generally, in dogs in European countries, seroprevalence appears to maintain a similar range to that observed in North America, at 1–12% [39,41,42,43]. It is worth remembering that seroprevalence in dogs is positively associated with incidence of borreliosis in humans [38]. Our values for prevalence in pet dogs from Poland and Ukraine (4–7%) are within the range, but higher than values obtained for stray dogs in Sofia, Bulgaria (0.6%; [42]) and lower than in military working dogs from Austria (11%; [44]). In previous studies in Poland, (sero)prevalence ranged likewise 4–11% [39,45].

Phylogenetic and molecular analyses allowed the identification of *Borrelia burgdorferi* s.s. in three positive dogs in the current study. This species is responsible for canine borreliosis in North America, in Europe [11] and has also been identified previously in dogs in North-Western Poland [7,33,46,47]. It is pathogenic for humans and responsible for development of Lyme arthritis. In three other studies from Poland, *Bo. afzelii* DNA has been detected in dogs, including a fatal case of myocarditis [9,45,48]. Additionally, *Bo. garinii* has been reported as the cause of canine borreliosis in Europe [7,11,49]. Interestingly, both *Bo. afzelii* and *Bo. garinii* appear to be more common in ticks in Poland than *Borrelia burgdorferi* s.s., in both natural and peri-urban areas [4], so the dominance of *Borrelia burgdorferi* s.s. in dogs may reflect its higher pathogenicity for these hosts. However, not all *Borrelia*-positive samples were successfully sequenced in the present study, so other species from *Borrelia burgdorferi* s.l. complex could have been also involved.

Our study confirmed that babesiosis due to *B. canis* is endemic in companion dogs in both Poland and Ukraine. Although there is a significant amount of published data on the occurrence of *B. canis* in dogs and *De. reticulatus* ticks from Poland [15,20,21], data on *B. canis* in ticks from Ukraine are scarce [50,51] but nevertheless confirm endemicity of this pathogen in the country. In the current study, the DNA of *B. canis* was identified in symptomatic dogs from Poland and Ukraine and our study is likely the first to provide data on occurrence of *B. canis* paralogs in Ukrainian dogs. Analysis of chromatograms revealed the occurrence of identical paralogs in dogs from Central Poland and Western Ukraine, providing evidence that geographical differences in their distribution are unlikely. The identified paralogs were in agreement with the results of an earlier initial study deciphering their occurrence in the 18S rDNA of *B. canis* in dogs from Poland [34].

The DNA of *B. canis* was also identified in a small percentage of ‘healthy’ dogs, both from Poland and Ukraine. Subclinical infections of *B. canis* have been previously observed in dogs, including military working dogs from Austria and in sled dogs from Poland [28,44]. However, in the latter study a much higher percentage of healthy dogs was found to be infected (25%) in comparison to the current study (4–5.4%). A lower prevalence of *B. canis* in healthy dogs may reflect better current awareness of the owners and veterinary practitioners and better prophylaxis against ticks [23,27].

Interestingly, we found several dogs with co-infections of *B. canis, Borrelia burgdorferi* s.l. and *Di. repens*. Co-infections with *B. canis* and *Di. repens* have been identified previously in numerous dogs from Central Poland [52], in dogs from Slovakia [53] and also in numerous dogs from Lithuania [24]. A triple co-infection of *B. canis, Borrelia burgdorferi* s.l. and *Di. repens* was also recently identified in a Malinois military dog from Austria [44] and co-infection with four pathogens, *Di. repens, A. phagocytophilum, Borrelia* spp. and *B. canis*, was reported in two dogs from Lithuania [24]. Co-infections may be the result of higher exposure of certain dogs to vectors, ticks and mosquitoes, but may also be associated with higher individual susceptibility of some individuals. In our previous study dogs with *Di. repens* infection appeared to be more susceptible to *B. canis* infection based on prevalence [52]. Co-infections, including with *Di. repens*, may become more common in dogs in central, eastern and NE Europe due to the continued spread of this nematode and *B. canis* in these regions of Europe [17,54]. 

## 5. Conclusions

*Babesia* spp. and *Borrelia burgdorferi* s.l. infections were not detected in any of the racing sled dogs sampled from seven countries (Poland, Lithuania, Latvia, Estonia, Belarus, Russia and Finland). In contrast, these pathogens were detected in pet dogs from Poland and Ukraine, including co-infections with *Di. repens*. Sequencing of PCR products enabled identification of *B. canis* and *Borrelia burgdorferi* s.s., the etiological agent of Lyme arthritis, in dogs. Furthermore, a wide occurrence of *B. canis* 18S rDNA paralogs was identified both in dogs from Poland and Ukraine. The study revealed endemic occurrence of vector-borne infections in dogs from Central Poland and Western Ukraine.

## Figures and Tables

**Table 1:** Prevalence of pathogens in groups of sampled dogs.

	Country	Pathogen	Sled Dogs (Healthy)	Pet Dogs (Healthy)	Pet Dogs with Babesiosis	Total No. of Dogs
The Baltic States	Lithuania	*Babesia* *Borrelia*	0/77 (0%) 0/77 (0%)	nd	nd	0/77 0/77
	Latvia	*Babesia* *Borrelia*	0/40 (0%) 0/40 (0%)	nd	nd	0/40 0/40
	Estonia	*Babesia* *Borrelia*	0/22 (0%) 0/22 (0%)	nd	nd	0/22 0/22
	Russia	*Babesia* *Borrelia*	0/11 (0%) 0/11 (0%)	nd	nd	0/11 0/11
	Finland	*Babesia* *Borrelia*	0/6 (0%) 0/6 (0%)	nd	nd	0/6 0/6
	Belarus	*Babesia* *Borrelia*	0/5 (0%) 0/5 (0%)	nd	nd	0/5 0/5
	Poland	*Babesia* *Borrelia*	0/95 (0%) 0/95 (0%)	4/74 (5.4%) 3/74 (4.1%)	68/68 (100%) 3/68 (4.4%)	72/237 6/237
	Ukraine	*Babesia* *Borrelia*	nd nd	4/105 (3.8%) 7/105 (6.7%)	41/50 (82%) 0/50 (0%)	45/155 7/155
	Total	*Babesia* *Borrelia*	0/256 (0%) 0/256 (0%)	8/179 (4.5%) 10/179 (5.6%)	109/118 (92.4%) 3/118 (2.5%)	117/553 13/553

nd—not done.

## Data Availability

The data are available from the corresponding author on reasonable request.

## Supplementary Materials

The following supporting information can be downloaded at: https://www.mdpi.com/article/10.3390/pathogens11050499/s1, Figure S1: Chromatograms representing different 18S rDNA paralogs.
