# Supplementary figures and images for: Tick-Borne Pathogens, Babesia spp. and Borrelia burgdorferi s.l., in Sled and Companion Dogs from Central and North-Eastern Europe
Source: Pathogens. 2022 Apr 21;11(5):499. doi: 10.3390/pathogens11050499 (PMC9144822; doi:10.3390/pathogens11050499)

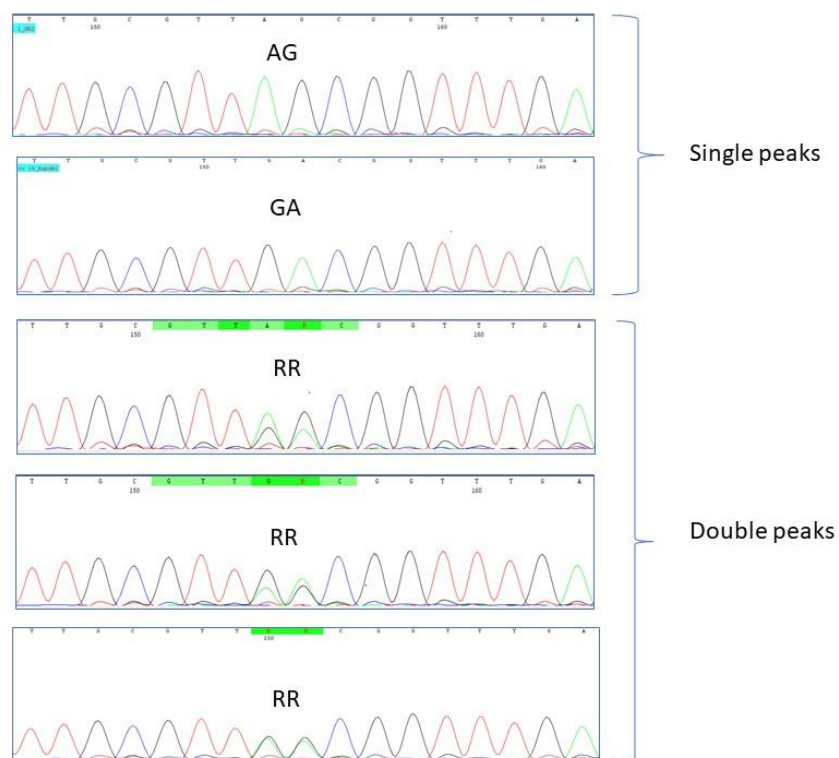

**Figure S1:** Chromatograms representing different 18S rDNA paralogs.

Supplement: Supplementary file 1 [file pathogens-11-00499-s001.zip › pathogens-1675076-supplementary.pdf]
